# Stereoacuity, Fusional Vergence Amplitudes, and Refractive Errors Prior to Treatment in Patients with Attention-Deficit Hyperactivity Disorder

**DOI:** 10.4274/tjo.galenos.2019.17802

**Published:** 2020-03-05

**Authors:** Irmak Karaca, Elif Demirkılınç Biler, Melis Palamar, Burcu Özbaran, Önder Üretmen

**Affiliations:** 1 Ege University Faculty of Medicine, Department of Ophthalmology, İzmir, Turkey; 2 Ege University Faculty of Medicine, Department of Child and Adolescent Psychiatry, İzmir, Turkey

**Keywords:** Attention-deficit and hyperactivity disorder, fusional vergence, stereoacuity

## Abstract

**Objectives:**

To evaluate stereoacuity, fusional vergence amplitudes, and refractive errors in patients with attention-deficit hyperactivity disorder (ADHD).

**Materials and Methods:**

Twenty-three patients who were newly diagnosed as having ADHD and had not started medication, and 48 children without ADHD were included. Retrospective data analysis of comprehensive eye examination, stereoacuity, and fusional vergence amplitudes of the patients were performed.

**Results:**

The mean age at ADHD diagnosis was 10.68±2.34 (7-16) years in the ADHD group (14 male, 9 female) and 12.23±2.16 (7-15) years in the control group (25 male, 23 female) patients (p=0.605). The mean stereoacuity was 142.14±152.65 (15-480) sec/arc in patients with ADHD and 46.3±44.11 (15-240) sec/arc in the control group (p<0.001). For ADHD patients, the mean convergence and divergence amplitudes at distance were 19.87±8.40 (6 to 38) prism diopter (PD) and -9.09±-4.34 (-4 to -25) PD, and 37.30±12.81 (14 to 70) PD and -13.13±-3.45 (-4 to -20) PD at near, respectively. The mean cycloplegic spherical equivalent was 1.06±1.13 (-1 to 4.63) diopter in ADHD patients, with 6 patients having significant refractive errors (hyperopia in 4 patients, astigmatism in 2 patients). There were no significant differences between groups in terms of spherical equivalents (p=0.358) or convergence and divergence amplitudes at distance (p=0.289 and p=0.492, respectively) or near (p=0.452 and p=0.127, respectively).

**Conclusion:**

Fusional vergence amplitudes did not present significant difference, while the mean value of stereoacuity was significantly lower in newly diagnosed ADHD patients prior to treatment.

## Introduction

Attention-deficit hyperactivity disorder (ADHD) is one of the most common neurodevelopmental disorders in children and adolescents. The prevalence of ADHD in developed countries is reported to be 2-18% among children between the ages of 6 and 17 years.^1,2^ ADHD is characterized by low attention, increased hyperactivity, impulsivity, and lack of control of inappropriate behaviors.^3^ In addition to quality of life, school performance is likely to be affected in patients with ADHD. In the literature, despite the variable results in studies associating visual dysfunction with school performance, there is a possible relationship between the symptoms of visual problems and ADHD-related behaviors.^4,5^

Brain imaging studies demonstrated delayed maturation in the brains of ADHD patients, with reduced striatal volume and differences in hippocampal, accumbens, and amygdala volumes compared to healthy controls. These findings along with dopamine and norepinephrine imbalance in the prefrontal cortex supported the deficits in emotional regulation, motivation, and memory in these patients.^6,7^ As the eyes are considered a continuation of central nervous system, the ocular system enables the evaluation of neurological changes and nervous system activation/inhibition.^8^ In patients with ADHD, binocular vision changes and oculomotor deficits such as convergence insufficiency^9,10^, accommodative dysfunction^11^, reduced stereoacuity^12^, and ametropia^13^ have been reported. Regarding underlying mechanisms, Poltavski et al.^14^ suggested a bidirectional relationship between attention and accommodation. However, the association between ADHD and oculomotor control changes such as accommodative dysfunction and convergence insufficiency is not clearly known yet. Additionally, there are several contradictory reports in terms of stereoacuity, refractive state, etc.^12,13^, and treatment status of the patients enrolled in those studies also varied. Therefore, this study aims to investigate stereoacuity, fusional vergence amplitudes, and cycloplegic refractive errors in newly diagnosed ADHD patients prior to medication.

## Materials and Methods

The charts of 23 consecutive patients who were newly diagnosed with ADHD according to DSM-IV criteria^3^ and had not yet received medication, and the data of 48 control patients of similar age and sex distribution who did not have ADHD and consecutively presented to the ophthalmology outpatient clinic for routine evaluation were retrospectively reviewed. The study was approved by the Institutional Ethics Review Board of Ege University and adhered to the precepts of the Declaration of Helsinki. Patients with congenital or acquired ophthalmic pathologies (such as optic nerve disease, glaucoma, cataract or additional media opacities, amblyopia, and strabismus), ophthalmic surgery history, and systemic or neuropsychiatric diseases other than ADHD were excluded. Stereoacuity measured with TNO Random-dot Stereo test (Lameris Intrumenten, Groeningen, Netherlands, 17^th^ Edition), fusional vergence amplitudes, presence of heterophoria with cover/uncover test, best corrected visual acuity (BCVA) according to Snellen scale, and spherical equivalent (SE) of refractive errors (Topcon KR-7000P) (Topcon Europe BV, Capellea/dIJssel, Netherlands) after cycloplegia (with 1% cyclopentolate hydrochloride) were recorded for all patients. As described in the literature^15^, fusional convergence and divergence amplitudes at distance (6 m) and near (33 cm) were measured by the same two examiners (I.K., E.D.B.) 3 times at 15-minute intervals with placement of the fixed horizontal prism bar (1D-40D) in front of an eye in all patients, while they fixated on an accommodative target. The base-out prism power was gradually increased for convergence and the base-in prism bar was gradually increased for divergence, and the patient was asked to identify the point at which the target image appeared to be doubled; this prism power was designated as the breakpoint. Patients with spectacles were tested with habitual optical correction in glasses or contact lenses. The mean value of the fusional vergence measurements were taken into account for the statistical analysis. Significant refractive errors were defined as an SE of myopia ≥0.5 diopter (D) or hyperopia ≥1.0 D. Significant astigmatism was defined as a level of ≥1.0 D and anisometropia, ≥1.0 D SE.^16^

### Statistical Analysis

The statistical analysis was performed using SPSS software for Windows version 15.0 (SPSS Inc, Chicago, Illinois, USA) and Microsoft Office Excel (Microsoft, Redmond, Washington, USA). For stereoacuity, all values were transformed to the logarithm of arc seconds.^17^ Statistical analyses were performed by independent t-test and chi-square test. A p value <0.05 was accepted as statistically significant.

## Results

The mean age of the subjects was 10.68±2.34 (range=7 to 16) years in patients with ADHD (14 male, 9 female) and 12.23±2.16 (range=7 to 15) years in the control group (25 male, 23 female). Anterior segment and fundus examinations were unremarkable and BCVA was 20/20 in both eyes of all subjects. No patient presented with restriction of eye movements, heterotropia, or anisometropia. The 22 patients (95.5%) in the ADHD group who were able to perform TNO Random-dot Stereo test had stereoacuity (at least 480 sec/arc) with full refractive correction. The mean and median values for stereoacuity were 142.14±152.65 (range=15-480) sec/arc and 60 sec/arc, respectively. The mean of convergence and divergence amplitudes at distance were 19.87±8.40 (range=6 to 38) prism diopter (PD) and -9.09±-4.34 (-4 to -25) PD, and at near 37.30±12.81 (14 to 70) PD, and -13.13±-3.45 (-4 to -20) PD, respectively. The SE following cycloplegia was 1.06±1.13 (range=-1 to +4.63) D. Thirteen patients were found to have significant refractive errors (hyperopia in 13 patients, astigmatism in 2 patients). Two patients who had hyperopia higher than 3.5 D were prescribed spectacles due to asthenopia and low BCVA. In the control group, all subjects were able to perform TNO Random-dot Stereo test and had stereoacuity with a mean of 46.3±44.11 (range=15 to 240) and the median of 30 sec/arc. The mean of convergence and divergence amplitudes at distance were 23.54±6.24 (range=14 to 36) and -9.67±-3.71 (range=-4 to -16) PD, and at near 38.21±8.13 (range=25 to 64) and -15.76±-3.52 (range=-12 to -25) PD, respectively. Following cycloplegia, the mean SE was 0.53±1.76 (range=-2.75 to 2.12) D, while 13 subjects had significant refractive errors (myopia in 6 patients, hyperopia in 7 patients, and astigmatism in 3 patients). There were no statistically significant differences between groups in terms of SE (p=0.358) or convergence and divergence amplitudes at distance (p=0.289 and p=0.492, respectively) or near (p=0.452 and p=0.127, respectively). Stereoacuity, fusional vergence amplitudes, and refractive status of the subjects are summarized in Table 1.

## Discussion

ADHD is thought to be associated with several negative outcomes, such as antisocial behaviors, social and peer problems, and psychiatric disorders later in life.^18,19^ Specifically, school performance, along with intellectual capacity, social abilities and occupational functions are impaired in patients with ADHD. In addition, ADHD is considered a major public health problem due to the considerable economic burden to families and community.^3,20,21^

A possible association between ADHD and visual problems was reported.^4,5^ In the literature, there are only a few studies revealing the relationship between ADHD and ocular abnormalities.^8,13,22,23^ Granet et al.^8^ retrospectively evaluated 266 children with convergence insufficiency and reported that 26 (21 male, 5 female) patients (9.8%) had ADHD either at the time of diagnosis or during the follow-up period. Among them, 20 (76.9%) patients were on medical treatment and 6 patients had never received treatment or had discontinued treatment. Additionally, a review of 176 ADHD patients who underwent ophthalmic evaluation revealed that 29 patients (15.9%) were diagnosed with convergence insufficiency based on their medical records. Thus, the authors suggested that convergence insufficiency should be investigated in patients with ADHD, despite the lack of causal relationship. Vergence is defined as simultaneous movements of eyes in opposite directions in order to have single binocular vision.^24^ Viewing through a range of prisms placed before the eyes in both a base-in and then base-out direction has long been used as a diagnostic measure of vergence and accommodation dysfunction.^25^ The capacity to see a single image via the base-out prisms without diplopia or blur is referred to as the fusional limit and determines the strength of convergence response, and vice versa.^26^ Therefore, fusional amplitudes have an important role in the maintenance of single binocular vision.^15^ For instance, low positive fusional limits, with the accompanying complaints of asthenopia, blur, or diplopia leading to frontal headaches following prolonged periods of near work, might be distracting and adversely affect school performance.^27,28,29^ Moreover, oculomotor dynamics are related to brain areas controlling attention and demonstrate sensitivity to alterations in attentional status.^14^ Animal models also indicated that the superior colliculus (SC), which constitutes the principal subcortical area involved in ocular control, participates in the regulation of near response, visual fixation, accommodation, and convergence.^30,31^ The SC is also linked to distractibility and proposed to be dysfunctional in ADHD.^32^Therefore, the evaluation of stereoacuity and fusional vergence amplitudes are of special importance in patients with ADHD. Gronlund et al.^12^ evaluated ADHD patients receiving medical treatment both before and 2 hours after stimulant use. They reported that the proportion of patients with stereoacuity ≥60 sec/arc was significantly higher in the ADHD population, independent of stimulant use. The rate of convergence near point <6 cm was significantly lower in ADHD patients before stimulant use, while there was no significant difference between the groups 2 hours after stimulant use. Fabian et al.^23^ compared 56 ADHD patients with 66 control subjects and did not find a statistically significant difference in terms of stereoacuity (41.5 sec/arc and 40.8 sec/arc, respectively; p=0.29) or fusional vergence amplitudes. They also noted that 15 patients (27%) were currently taking methylphenidate treatment. On the other hand, Fabian et al.^23^ did not report convergence insufficiency in any of their patients. Despite the near point of convergence being significantly lower in patients with ADHD [5.3 (range, 3 to 15) and 4.1 (range, 2 to 10); p=0.002], this did not reveal any clinical importance, since these values were <6 cm. In the present study, although all patients with ADHD had stereoacuity of at least 480 sec/arc, the mean value of stereoacuity in these patients was significantly lower compared to the control group. In addition, there was no significant difference in terms of fusional vergence amplitudes. The limitation of the present study is the lack of data regarding near point of convergence. However, convergence insufficiency was not present in any of the patients in terms of symptoms or decreased positive fusional vergences (both at the near point). These findings are also consistent with the report of Fabian et al.^23^ In contrast, in the present study, all patients were newly diagnosed with ADHD and had not previously received any medication, which eliminates the confounding effect of medication on these parameters. On the other hand, Bennett et al.^33^ suggested that some of the medications used in the treatment of ADHD might worsen convergence insufficiency and certain drugs may lead to blurry vision due to difficulty in accommodation. Herein, convergence insufficiency was thought to be a comorbidity rather than a disease-related problem, since fusional vergence amplitudes were similar in both groups. Also, when the prevalence of convergence insufficiency is believed to be as low as 2.25-13% in school-age children^34,35^, the sample size of our study does not seem to be adequate to make a definite conclusion. Therefore, more precise results will be achieved with larger prospective studies which will also evaluate the follow-up of ADHD patients. Another potential bias might have occured in relation to the examiner, who was only partially blinded to which children were in which group.

Refractive changes in patients with ADHD were previously investigated and no statistically significant difference was determined as compared to controls (Table 2).^11,23^ Larranaga-Fragoso et al.^36^ reported that in patients with ADHD, SE before and after cycloplegia did not differ significantly during 9 months of follow-up. As compared with the literature, they suggested that methylphenidate treatment does not affect refraction in children with ADHD. Besides, the relationship between hyperopia and learning difficulties has not yet been clarified. Although some studies reported that hyperopia is associated with different developmental problems and low school performance^37^, some studies did not reveal such an association.^38,39^ However, in most studies measurements were obtained without cycloplegia. In hyperopia, unlike myopia, diagnosis may be delayed due to clear visualization through excessive accommodation at near distances. Excessive accommodation may also result in asthenopia, distractibility, hyperactivity, and learning difficulties.^39,40^ Additionally, it is stated that hyperopia >3.5 D increases strabismus and visual acuity problems and is accepted as an amblyogenic risk factor.^40^ In the present study, SE did not show a significant difference between groups. However, the prevalence of hyperopia was higher in patients with ADHD, whereas myopia was more commonly observed in control subjects (Table 2). This also suggests that visual problems may be associated with disorders such as ADHD.

## Conclusion

In conclusion, this study showed that fusional vergence amplitudes did not differ significantly, whereas the mean value of stereoacuity was significantly lower in newly diagnosed and unmedicated ADHD patients. Despite the similar fusional vergence amplitudes, it is possible that low stereoacuity in patients with ADHD may suggest the lack of adequate attention while performing TNO random-dot stereo test. Nevertheless, it could be beneficial for children with vision problems to be examined for signs and symptoms of ADHD and vice versa.

## Figures and Tables

**Table 1:**
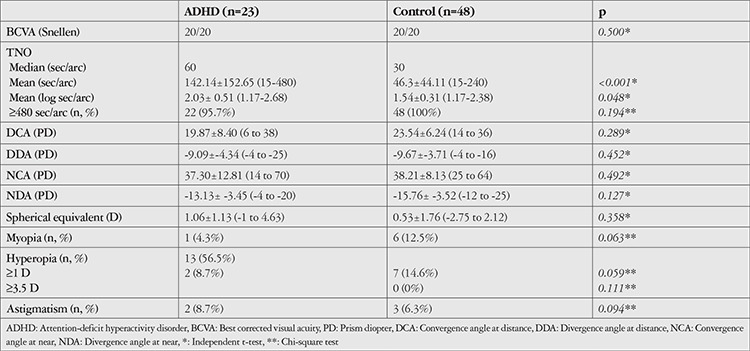
Stereoacuity, fusional vergence amplitudes, and refractive status of the children

**Table 2:**
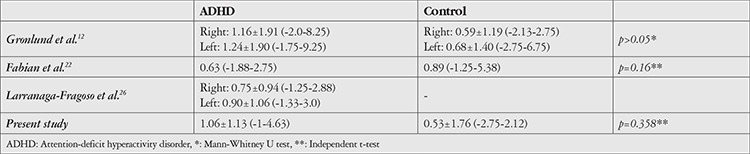
Spherical equivalents after cycloplegia in patients with ADHD in the literature
